# Case Report: mTOR inhibitor treatment for epithelioid angiomyolipoma harboring biallelic *TSC2* mutations

**DOI:** 10.3389/fonc.2026.1735690

**Published:** 2026-01-30

**Authors:** Shiori Ishikawa, Kota Ouchi, Shonosuke Wakayama, Yuki Kasahara, Keigo Komine, Hiroo Imai, Ken Saijo, Yuto Yamazaki, Masanobu Takahashi, Hidekazu Shirota, Hisato Kawakami

**Affiliations:** 1 Department of Clinical Oncology, Tohoku University Graduate School of Medicine, Sendai, Japan; 2 Department of Medical Oncology, Tohoku University Hospital, Sendai, Japan; 3 Department of Pathology, Tohoku University Hospital, Sendai, Japan

**Keywords:** angiomyolipoma, CGP, eAML, everolimus, *TSC2*

## Abstract

**Introduction:**

Angiomyolipoma (AML) is a mesenchymal tumor composed of blood vessels, smooth muscle, and adipose tissue, and is generally considered benign. However, epithelioid angiomyolipoma (eAML) is a rare and aggressive variant with metastatic potential. Molecular characterization utilizing the tuberous sclerosis complex (TSC)–mTOR pathway is beneficial in advanced disease. This report describes the clinical course, histopathological findings, and molecular analysis of a patient with metastatic eAML.

**Methods:**

A 59-year-old Japanese man with no personal or family history of tuberous sclerosis (TSC) was admitted to the hospital with gradually worsening back pain and initially diagnosed with clear cell renal cell carcinoma (ccRCC). He underwent nephrectomy, followed by hepatic recurrence treated with pazopanib and subsequent axitinib. Both were discontinued due to intolerance, and the two remaining liver metastases were surgically resected. Histopathological examination of the resected lesions revealed eAML. After several recurrences and resections, unresectable hepatic and pulmonary metastases eventually developed.

**Results:**

Comprehensive genomic profiling (CGP) using the resected liver metastasis specimen identified two somatic *TSC2* mutations: a frameshift mutation (p. P677fs*21; variant allele frequency [VAF] 0.0789) and a nonsense mutation (p. S1469*; VAF 0.0736), suggesting biallelic loss of *TSC2.* Based on these findings, everolimus, a mammalian/mechanistic target of rapamycin (mTOR) inhibitor, was recommended, which markedly reduced the size of the metastatic lesions and was continued for 24 months until disease progression without severe adverse events.

**Discussion:**

This case suggests that CGP can help identify actionable alterations in eAML, such as *TSC2* mutations, to guide personalized therapy with mTOR inhibitors.

## 1 Introduction

Angiomyolipoma (AML) is one of the most common benign solid renal tumors (1), with an estimated prevalence of 1–3% among solid renal tumors (2) and 0.2–0.3% in the general population (3). Approximately 20–30% (4) of AML cases are associated with tuberous sclerosis complex (TSC), a hereditary disorder characterized by multiple hamartomas (5). AML comprises blood vessels, smooth muscle, and adipose tissue, and is generally regarded as benign (6). In contrast, the rare variant epithelioid angiomyolipoma (eAML) is characterized by predominant epithelial cells with clear eosinophilic cytoplasm and nuclear atypia (7), and often exhibits a malignant phenotype, including metastasis (8). Although renal eAML typically shows resistance to radiotherapy, conventional chemotherapy, and molecular targeted therapies (9), recent advances in cancer genomics have enabled the identification of effective treatments for rare malignancies (10, 11). Herein, we report a case of eAML successfully treated with matched therapy, as suggested by comprehensive genomic profiling (CGP).

## 2 Case description

**Figure 1:**
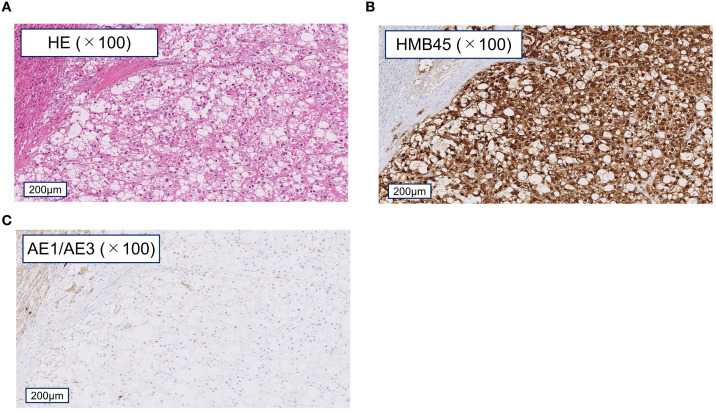
Tumor tissue specimens from liver metastasis. Microscopic images of liver metastasis specimens. **(A)** Hematoxylin and eosin staining showing predominantly epithelioid tumor cells with enlarged nuclei and abundant eosinophilic cytoplasm. **(B)** Immunohistochemical staining demonstrating diffuse positivity for human melanoma black 45 (HMB-45). **(C)** Tumor cells negative for cytokeratin AE1/AE3. Scale bar: 200 µm.

A 59-year-old Japanese male was admitted with gradually worsening back pain. He had no relevant medical history and was not taking regular medications. He had a 39-year history of smoking 20 cigarettes per day and was a social drinker. Although there was a family history of malignant tumors (**Supplementary Figure S1**), there was no personal or family history of TSC. Computed tomography (CT) revealed a low-density lesion in the left kidney. A renal biopsy in May 2014, followed by left nephrectomy in July 2014, led to a diagnosis of pT2acN0M0 (Stage II) clear cell renal cell carcinoma (ccRCC). No postoperative adjuvant therapy was administered. Two years later, two hepatic nodules appeared and were clinically diagnosed as ccRCC liver metastases. Pazopanib was initiated in May 2016, followed by axitinib in September 2016; however, both were discontinued due to severe adverse events. In August 2017, the two remaining liver metastases were surgically resected. Histopathological examination revealed a tumor predominantly comprised of epithelioid cells with enlarged nuclei and abundant eosinophilic cytoplasm. Immunohistochemistry revealed diffuse positivity for human melanoma black 45 (HMB-45; **Figure 1B**) and negativity for cytokeratin AE1/AE3 (**Figure 1C**), leading to a diagnosis of eAML. Retrospective review of the initial renal tumor resulted in revision of the initial diagnosis to eAML. The patient was managed by active surveillance. From May to July 2019, recurrent liver metastases occurred three times and were surgically resected on each occasion. Eventually, unresectable hepatic and pulmonary metastases developed. Doxorubicin was initiated following the standard chemotherapy regimen for sarcomas in September 2019. However, treatment was discontinued 10 days after the first dose because of a grade 3 abdominal infection. Given the lack of established standard therapy for eAML, CGP was pursued to identify potential treatment options.

**Figure 2:**
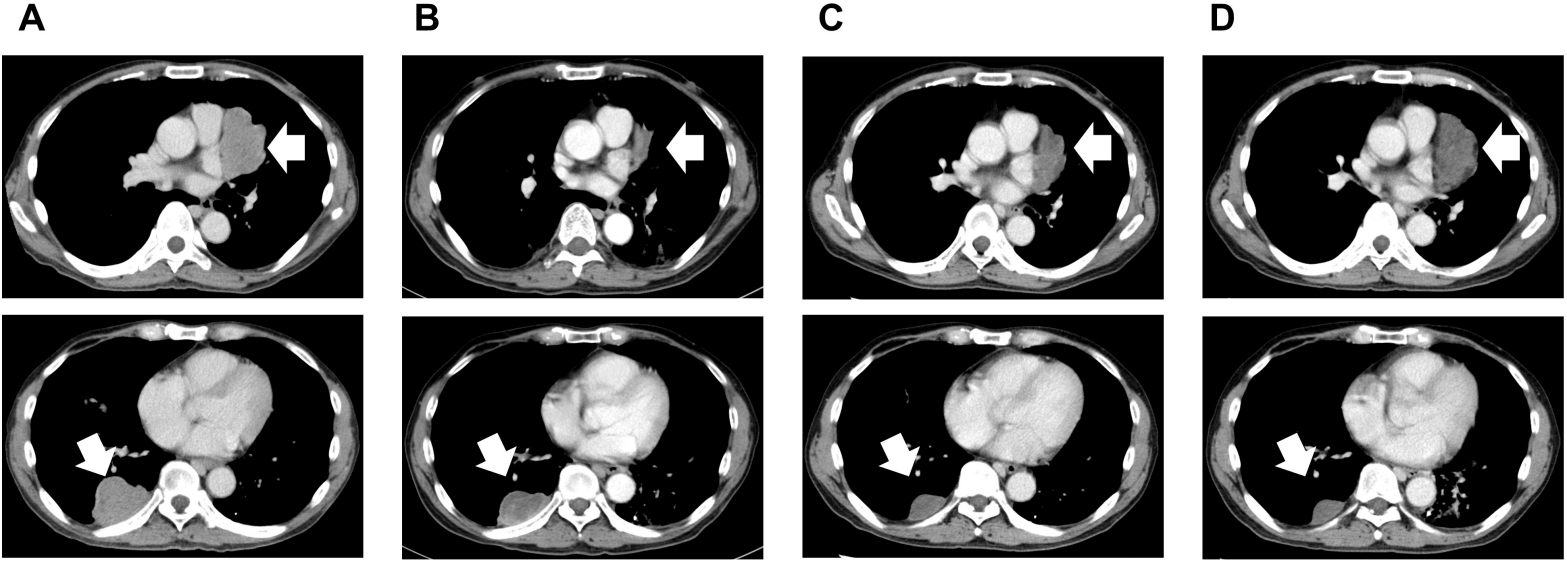
Computed tomography of metastatic lesions in the thoracic cavity before and during everolimus administration. Contrast-enhanced computed tomographic images at **(A)** the initiation of everolimus treatment and at 4 months **(B)**, 1 year **(C)**, and 2 years **(D)** after treatment initiation. Arrows indicate the tumor lesions.

CGP using FoundationOne^®^ CDx assay (Foundation Medicine, Cambridge, MA, USA) on the specimen from the initial hepatic resection identified two *TSC2* mutations: a nonsense mutation (c.4406C>G, p.S1469*; allele frequency, 0.0789) and a frameshift (c. 2029_2042delCCCGCCGTGCGGCT, p.P677fs*21; allele frequency, 0.0736). No other pathogenic alterations were detected (**Table 1**). Both *TSC2* variants were predicted loss-of-function alterations, consisting of one nonsense mutation (p.S1469*) and one frameshift mutation (p.P677fs*21), each introducing a premature stop codon upstream of the GTPase-activating protein (GAP) domain of tuberin. In silico pathogenicity prediction using the Combined Annotation Dependent Depletion (CADD) (12) framework indicated a high deleteriousness score for a frameshift (c.4406C>G, p.S1469*) (PHRED > 30), supporting their truncating and pathogenic nature. Based on these findings, the Molecular Tumor Board recommended treatment with a mammalian target of rapamycin (mTOR) inhibitor. Everolimus was orally administered at a dose of 10 mg/day in January 2020. Both hepatic and pulmonary metastases showed marked shrinkage within 4 months of treatment initiation (**Figure 2**). During everolimus therapy, grade 1 interstitial pneumonia and diarrhea appeared but resolved spontaneously without therapeutic intervention or treatment discontinuation. Approximately 1 year after everolimus initiation, the central pulmonary lesion showed radiological progression and was considered the most threatening site of disease. Local treatment options, including radiotherapy and surgical resection, were discussed in the multidisciplinary team; however, they were not pursued due to the multifocal distribution of metastases and the patient’s general condition. Given ongoing systemic disease control, everolimus was continued and maintained for a total of 24 months until the development of hepatic and pulmonary metastases. The patient subsequently opted for palliative care and was followed up continuously for 3 months. A concise overview of the patient’s course is presented in **Table 2**.

**Table 1:** The list of gene mutations detected by comprehensive genomic profiling.

Gene	Ref seq	Variant type	Exon	Mutation (AA)	Mutation (DNA)	Allele frequency
*FANCA* ^†^	NM_000135	Missense	n/a	p.R1409Q	c.4226G>A	0.4691
*TSC2*	NM_000548	Nonsense	n/a	p.S1469*	c.4406C>G	0.0789
*ROS1* ^†^	NM_002944	Missense	n/a	p.Y338C	c.1013A>G	0.5198
*ABL1* ^†^	NM_005157	Deletion	n/a	p.S882del	c.2643_2645delCTC	0.4974
*TSC2*	NM_000548	Frame Shift	n/a	p.P677fs*21	c.2029_2042delCCCGCCGTGCGGCT	0.0736
*NF1* ^†^	NM_001042492	Missense	n/a	p.L1183M	c.3547C>A	0.4782
*VEGFA* ^†^	NM_001025366	Missense	n/a	p.A123T	c.367G>A	0.513

AA, amino acid; † variant of unknown significance; n/a, not available.

**Table 2:** Abbreviated presentation of the patient’s course.

April, 2014	Admission with back pain
July, 2014	Nephrectomy, with diagnosis of ccRCC
May, 2016	Pazopanib administration
September, 2016	Axitinib administration
August, 2017	Hepatectomy, with diagnosis of eAML
May 2019 – July 2019	Surgical resection of liver metastases three times
September 2019	Doxorubicin administration to unresectable metastases
October 2019	CGP test
November 2019	Recommendation of an mTOR inhibitor
January 2020	Everolimus administration
March 2020	Partial response
February 2022	Progressive disease and palliative care

## 3 Discussion

eAML is distinct from classic AML (13) in that it comprises epithelioid tumor cells exhibiting pleomorphism and nuclear atypia, often accompanied by microscopic hemorrhage, cysts, necrosis, and minimal adipose tissue. These features mimic those of ccRCC (14), further delaying diagnosis, as in our case. Given the substantial histopathological overlap between eAML and clear cell renal cell carcinoma (ccRCC), an important clinical consideration is whether a more precise initial diagnosis would have influenced postoperative management. In recent years, adjuvant everolimus has been investigated in patients with high-risk ccRCC, most notably in the EVEREST phase III trial (15). Although this study suggested a potential benefit in selected high-risk subgroups, the overall efficacy of everolimus in the adjuvant setting was modest, underscoring the need for a cautious and individualized approach. Accordingly, even if the initial histopathological diagnosis had been established earlier, routine use of adjuvant everolimus would not have been readily justified based on currently available evidence. While *TSC2* alterations represent a biologically compelling therapeutic target in renal neoplasms, the role of everolimus as adjuvant therapy remains limited and should be considered on a case-by-case basis rather than as standard practice.

eAML is frequently associated with TSC, with 20–30% of cases occurring in this context, primarily due to germline mutations in *TSC1* or *TSC2* (4). In contrast, sporadic eAML commonly harbor somatic mutations in *TSC1* or *TSC2* (16), and biallelic loss of these genes has been reported in 57% of cases (17). Although the therapeutic efficacy of mTOR inhibitors has been reported in TSC-associated AML, evidence supporting their use in sporadic AML remains limited (9, 18, 19). The present case was considered sporadic because there was no family history or clinical findings suggestive of TSC, such as hamartomas. However, the absence of clinical diagnostic criteria does not exclude TSC, as mild or atypical forms without characteristic manifestations have been reported (20). Therefore, this patient met the criteria for CGP for pathogenic germline variants, including *TSC2*, to clarify whether the detected alterations were purely somatic or reflected an underlying TSC spectrum disorder.

In renal angiomyolipoma and related perivascular epithelioid cell tumors, biallelic loss of *TSC1/TSC2* is considered the primary and sufficient driver of tumorigenesis rather than a passenger alteration. Whole-exome sequencing studies have demonstrated that most lesions harboring *TSC1/TSC2* inactivation lack additional recurrent oncogenic mutations, highlighting the central role of the TSC complex in tumorigenesis (16). In our case, the presence of two truncating *TSC2* mutations with similar VAF (variant allele frequencies) and the absence of other pathogenic driver alterations strongly support *TSC2* as the driver event in eAML. The patient’s eAML harbored two *TSC2* variants (VAF 0.0789 and 0.0736) present at relatively low frequencies, likely reflecting low tumor purity or stromal admixture. Pathological review estimated tumor cellularity of the analyzed specimen to be approximately 15%, with substantial stromal and inflammatory cell admixture. The relatively low VAFs are therefore compatible with clonal events in a sample with limited tumor purity rather than subclonal mutations. The similar VAFs suggest that these variants exist within the same tumor clone. Although phasing information is not available to confirm whether the variants are in trans, the comparable VAFs and the known biology of TSC2-driven tumorigenesis strongly support the likelihood of biallelic loss. A limitation of our genomic analysis is the inability to directly determine the phase (in trans vs. in cis) of the two *TSC2* mutations. Thus, biallelic inactivation is inferred from the variant pattern, tumor histology, and the known biology of eAML. Whether these *TSC2* mutations result in complete loss of function or a hypomorphic form of tuberin remains an important biological question. Previous functional analyses have demonstrated that some *TSC2* variants can partially retain mTOR regulatory function, leading to hypomorphic phenotypes (21). However, in our case, both the nonsense and the frameshift mutations introduce early stop codons and are expected to abolish the GAP activity of the TSC1/TSC2 complex. Thus, it is more likely that these variants cause near-complete loss of tuberin function rather than a hypomorphic state.

The TSC1/TSC2 complex functions as a critical negative regulator in mTOR complex 1 (mTORC1), functioning as a GAP for the small GTPase Rheb. Loss of *TSC1* or *TSC2* disrupts this inhibitory complex, resulting in constitutive *mTORC1* activation, enhanced protein synthesis, and uncontrolled cell growth. Consequently, inactivation of *TSC1/TSC2* is a central event in the pathogenesis of TSC-associated and sporadic perivascular epithelioid cell tumors (PEComas), including eAML, and provides a strong mechanistic basis for the use of mTOR inhibitors in these tumors (22). Currently, standard chemotherapeutic options for the treatment of metastatic eAML are lacking. However, mTOR pathway blockade could be potent, given that *TSC2* loss-of-function drives the mTOR pathway, resulting in eAML development (23). Several case reports and case series have reported the response of eAML to mTOR inhibitors, such as everolimus (24) and sirolimus (18). A randomized controlled trial with long-term extension demonstrated that everolimus effectively reduces renal AML volume in patients with TSC or sporadic lymphangioleiomyomatosis, with sustained responses and manageable adverse events over extended follow-up (25). These findings support the role of mTOR inhibition in tumors driven by *TSC2* loss, providing a clinical context for the transient benefit observed in our case. Overall, the patient experienced a durable but ultimately temporary clinical benefit from everolimus, with marked tumor shrinkage followed by progression after approximately 2 years. This pattern aligns with previous reports of eAML or other malignancies harboring *TSC2* mutations, in which mTOR inhibitors frequently induce partial responses or long-lasting stable disease but are eventually followed by acquired resistance. Compared with published cases, the duration of benefit in our patient appears to be within the upper range of reported responses, suggesting that biallelic *TSC2* inactivation together with additional alterations (such as *NF1*) may initially confer high mTOR dependency, whereas subsequent treatments and clonal evolution might contribute to a more aggressive biology at relapse.

The efficacy of everolimus has been demonstrated in malignant neoplasms harboring biallelic *TSC2* mutations (26, 27). Previous studies in patients with TSC-associated renal lesions (25) have demonstrated that mTOR pathway activation is a key driver of tumor growth and that mTOR inhibition can reduce tumor burden and improve renal outcomes, providing a biological rationale for targeting mTOR signaling in renal neoplasms with *TSC1/TSC2* inactivation, including eAML. A prospective basket trial evaluated everolimus in patients with advanced solid tumors harboring *TSC1*, *TSC2*, or mTOR mutations (28). Although the overall response rate was modest, exploratory analyses suggested that tumors with concurrent biallelic *TSC2* inactivation and *NF1* mutations, as well as tumors with perivascular epithelioid cell tumors (PEComa)-like histologic features, might derive enhanced clinical benefit from mTOR inhibition. In our case, the co-occurrence of *NF1* and biallelic *TSC2* alterations—similar to the molecular constellation observed in that study—together with epithelioid morphology reminiscent of PEComa-spectrum tumors may partly explain the transient clinical benefit observed with everolimus, despite the eventual development of resistance. Further investigation in larger cohorts is warranted to validate the predictive value of such co-alteration profiles. In the present case, two truncating *TSC2* variants with similar VAF were identified, strongly suggesting somatic biallelic loss of *TSC2* within the same tumor cell population. Notably, comprehensive genomic profiling did not reveal any other clearly established driver mutations, further supporting the role of *TSC2* alterations as the primary oncogenic driver in this case. Taken together, eAML harboring biallelic *TSC2* mutations may be sensitive to mTOR inhibitors, as observed in our case.

To complement our single-patient observation, we queried the Center for Cancer Genomics and Advanced Therapeutics database for additional cases of eAML treated with mTOR inhibitors. Four patients with eAML were identified (#1–#4, **Supplementary Table S1**). Two harbored *TSC2* mutations (#2 and #4). mTOR inhibitors were administered to two patients (#1 and #2). Patient #2, carrying a *TSC2* short deletion, achieved stable disease, whereas Patient #1 achieved a partial response despite lacking a *TSC2* mutation. These findings suggest that *TSC2* alterations may serve as a useful biomarker, although sensitivity to mTOR inhibition is not necessarily exclusive to *TSC2*-mutated tumors.

## 4 Conclusion

Our findings suggest that CGP can guide personalized therapy for eAML for identifying actionable *TSC2* mutations for which mTOR inhibitors may offer meaningful clinical benefits.

## Data Availability

The datasets presented in this study can be found in online repositories. The names of the repository/repositories and accession number(s) can be found in the article/Supplementary Material.
