# Supplementary material for: Case Report: mTOR inhibitor treatment for epithelioid angiomyolipoma harboring biallelic TSC2 mutations
Source: Front Oncol. 2026 Jan 30;16:1735690. doi: 10.3389/fonc.2026.1735690 (PMC12900712; doi:10.3389/fonc.2026.1735690)
Supplement: Supplementary file 1 [file Supplementaryfile1.docx]

**Supplementary Material**

**Figure S1: Family history of the patient.**

Male and female family members are indicated by squares and circles, respectively. Symbols are in black for individuals with malignant tumors, with details provided outside the column. Age at death is indicated with “d.” placed below the symbol. The patient is indicated by “P” (proband) in the lower left corner of the symbol.


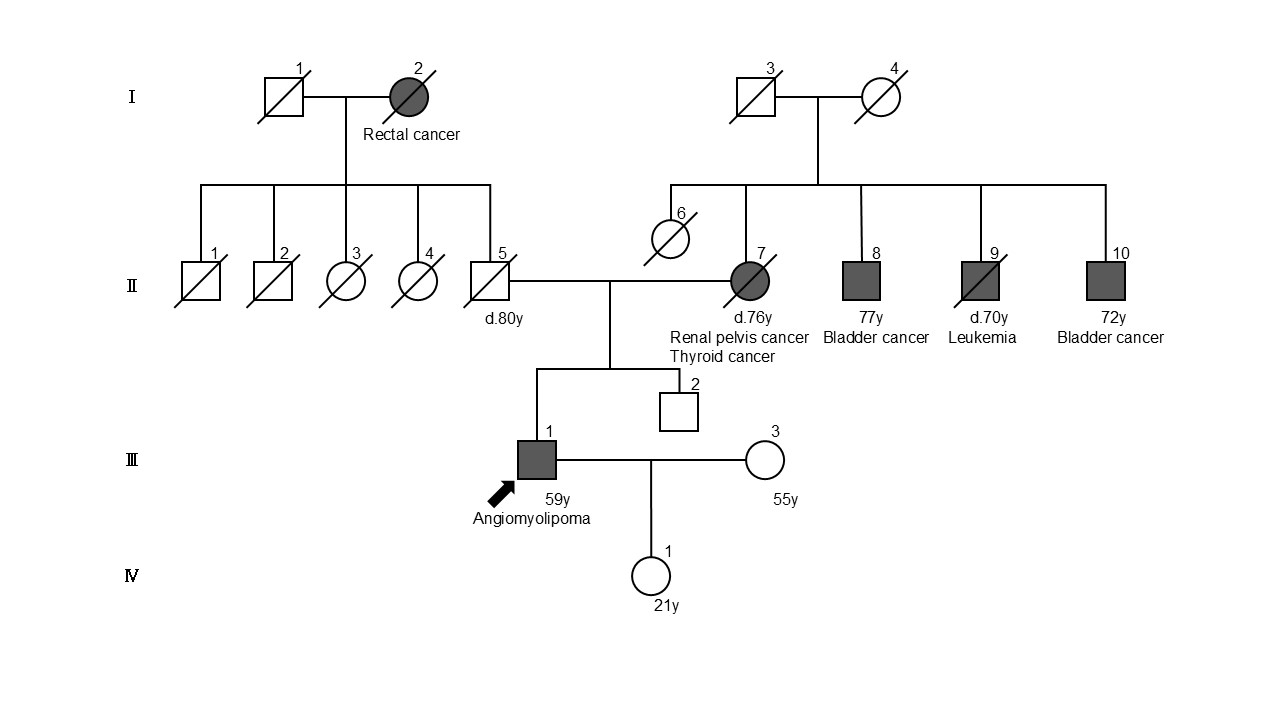


**Table S1:** **Profiles of eAML patients registered in the C-CAT database**

| Patient number | Sex | Age | Pathological diagnosis | Germline mutation  (VAF) | *TSC2* mutation  (VAF) | mTOR inhibitor | Best overall response |
| --- | --- | --- | --- | --- | --- | --- | --- |
| 1 | F | 38 | eAML | *RB1* R621S (0.520) *TP53* D49H (0.44) | none | Everolimus | PR |
| 2 | F | 65 | eAML | none | c.3251_3284+18del52  (0.19) | Temsirolimus | SD |
| 3 | M | 66 | eAML | none | none | none | none |
| 4 | F | 45 | eAML | none | c.5160+2_5160+3delTG  (0.1859) | none | none |
| our case | M | 59 | eAML | none | p.P677fs*21  (0.0789)  p.S1469*  (0.0736) | Everolimus | PR |
| AML, angiomyolipoma; eAML, epithelioid angiomyolipoma; C-CAT, Center for Cancer Genomics and Advanced Therapeutics; mTOR, mammalian/mechanistic target of rapamycin; NE, not evaluated; PR, partial response; SD, stable disease; VAF, variant allele frequency.  *TSC2* mutations were underlined. Missing data were indicated as “none”. | | | | | | | |
